# Immune thrombocytopenia associated with lymph node tuberculosis: a case report

**DOI:** 10.1590/0037-8682-0072-2023

**Published:** 2023-07-24

**Authors:** Juliano Córdova Vargas, Patrícia Nunes Bezerra, Murilo Sequeira, Gabriella Haydée de Oliveira, Carlos Eduardo Pegolo, Lucas Fiore, Nelson Hamerschlak, Paulo Roberto Abrão Ferreira

**Affiliations:** 1 Américas Oncologia e Hematologia, Departamento de Hematologia, São Paulo, SP, Brasil. Américas Oncologia e Hematologia Departamento de Hematologia São Paulo SP Brasil; 2 Hospital Samaritano Higienópolis, Departamento de Hematologia, São Paulo, SP, Brasil. Hospital Samaritano Higienópolis Departamento de Hematologia São Paulo SP Brasil; 3 Hospital Metropolitano da Lapa, Departamento de Hematologia, São Paulo, SP, Brasil. Hospital Metropolitano da Lapa Departamento de Hematologia São Paulo SP Brasil; 4 Centro Universitário São Camilo, Faculdade de Medicina, São Paulo, SP, Brasil. Centro Universitário São Camilo Faculdade de Medicina São Paulo SP Brasil; 5 Hospital Metropolitano da Lapa, Departamento de Radiologia, São Paulo, SP, Brasil. Hospital Metropolitano da Lapa Departamento de Radiologia São Paulo SP Brasil; 6 Hospital Samaritano Higienópolis, Departamento de Radiologia, São Paulo, SP, Brasil. Hospital Samaritano Higienópolis Departamento de Radiologia São Paulo SP Brasil; 7 Hospital Israelita Albert Einstein, Departamento de Hematologia, São Paulo, SP, Brasil. Hospital Israelita Albert Einstein Departamento de Hematologia São Paulo SP Brasil; 8 Universidade Federal de São Paulo, Departamento de Infectologia, São Paulo, SP, Brasil. Universidade Federal de São Paulo Departamento de Infectologia São Paulo SP Brasil

**Keywords:** Immune thrombocytopenia, Lymph node tuberculosis, Tuberculosis

## Abstract

Extrapulmonary tuberculosis associated with immune thrombocytopenia (ITP) is extremely rare. A likely association between ITP and pulmonary and lymph node tuberculosis was reported in a 29-year-old male patient. His platelet count decreased to 4,000/µL. Chest tomography revealed mediastinal adenomegaly, lymph node clusters in the aorta, and consolidation in the left upper lung lobe. Immunoglobulin and methylprednisolone were administered intravenously. The histopathology of the left upper lung lobe confirmed tuberculosis. The rifampicin/isoniazid/pyrazinamide/ethambutol regimen was initiated, and the corticosteroids were tapered off. This case suggests an association of tuberculosis with ITP, since the platelet count effectively normalized after tuberculosis treatment.

## INTRODUCTION

Tuberculosis is an infectious disease that can affect organs outside the respiratory system^1^^,^^2^. It may present as extrapulmonary lymph node tuberculosis. Of the 7,174 cases of tuberculosis reported in the US in 2020, extrapulmonary tuberculosis was the only form of the disease in 21% of cases, while pulmonary and extrapulmonary involvement were concomitantly present in 78.9% of cases^3^^,^^4^. Some risk factors for extrapulmonary tuberculosis include co-infection with viruses such as the human immunodeficiency virus (HIV), immunosuppression associated with age, and the use of pharmaceutical drugs. 

Immune thrombocytopenia (ITP) is a hematological autoimmune disease characterized by a decrease in platelet count due to the destruction of platelet cells, particularly in the spleen. In the absence of a secondary disorder or underlying cause, the condition is referred to as primary ITP of an unidentified cause. When it is secondary to other conditions, treatment of the underlying condition constitutes a crucial step in treating ITP^3^^,^^4^.

ITP involves an increase in platelet destruction in the peripheral blood and spleen, and a reduction in platelet production in the bone marrow. Several chronic and infectious diseases may cause secondary ITP in adults^5^. In ITP, the production of autoantibodies may result from the reaction of CD4-positive helper T cells with platelet surface glycoproteins and may also involve co-stimulatory interactions between CD40 and CD40L^5^^,^^6^. Although this is the most probable mechanism, antiplatelet antibodies are not found due to poor sensitivity^,^^8^. Consequently, antiplatelet antibody assays are not recommended for the diagnosis and management of ITP^2^^,^^5^.

The association of extrapulmonary tuberculosis with ITP is extremely rare^9^^,^^10^. Weber et al. reported on such a case and reviewed 50 cases of tuberculosis-associated ITP published between 1964 and 2016^11^. Most of the cases reviewed consisted of pulmonary tuberculosis, with only four cases of lymph node tuberculosis.

A literature review was conducted in the PubMed, Medline, and Lilacs databases using the search terms *extrapulmonary tuberculosis*, *lymph node* and *immune thrombocytopenia*. Using these criteria, 12 articles were identified in the LILACS database, 10 in MEDLINE and 10 in PubMed. After excluding articles in which no association between ITP and extrapulmonary tuberculosis was reported, 10 articles remained^1^^-^^3^^,^^5^^,^^6^^,^^7^^,^^8^^-^^12^.

The articles indicated that extrapulmonary tuberculosis is less common and more heterogenous, with ITP as a possible immune-mediated manifestation. The incidence of lymph node tuberculosis has increased in recent years and now corresponds to 25-60% of all cases of extrapulmonary tuberculosis. 

This report describes a case of ITP presumed to have resulted from tuberculosis with pulmonary and lymph node presentations in a 29-year-old male patient. This case is relevant and particularly intriguing because it involved lymph node tuberculosis in an immunocompetent patient, which resulted in secondary ITP. Clinical improvement and laboratory tests showing stable values were achieved only after treatment of the primary condition of tuberculosis.

## CASE REPORT

**FIGURE 1:**
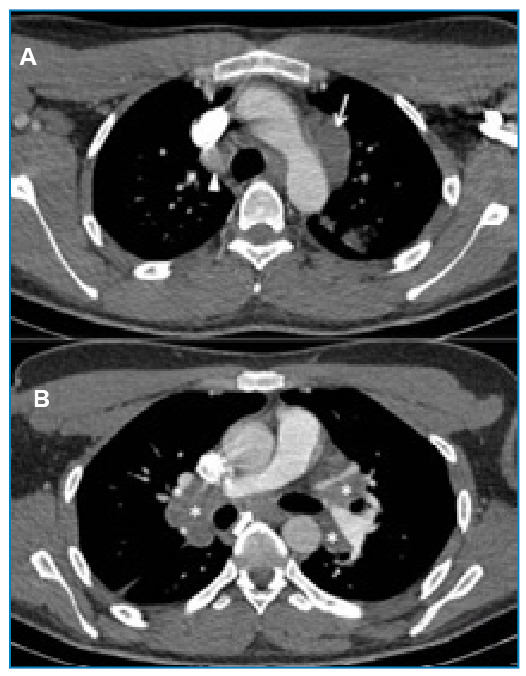
Chest tomography with the affected lymph nodes marked.

A 29-year-old man presented to the hospital with fever and night sweats for 30 days. The patient recently underwent videolaparoscopic cholecystectomy (hospital stay: September 10-11, 2019). In October 2019, a platelet count of 20,000/µL was the only blood count abnormality. Chest tomography revealed mediastinal adenomegaly, with some lymph nodes forming 2.9 x 2.5 cm clusters in the aorta and alveolar consolidation in the left upper lung lobe (Figure 1). Serology for hepatitis B, hepatitis C, and HIV was negative. 

Bronchoscopy with bronchoalveolar lavage yielded negative results for acid-fast bacilli and fungal and bacterial pathogens. The culture for *M. tuberculosis* was negative. Rapid molecular detection tests were not conducted. A liquid biopsy of the lavage fluid could not be performed; therefore, an open biopsy was conducted with resection of the para-aortic lymph node and adjacent pulmonary segmentectomy. 

Since platelet count fell further to 4,000/µL and was associated with disseminated ecchymoses and petechiae, intravenous immunoglobulin was initiated at a dose of 0.4 g/kg/day for 5 days, together with 1 mg/kg methylprednisolone. 

**FIGURE 2:**
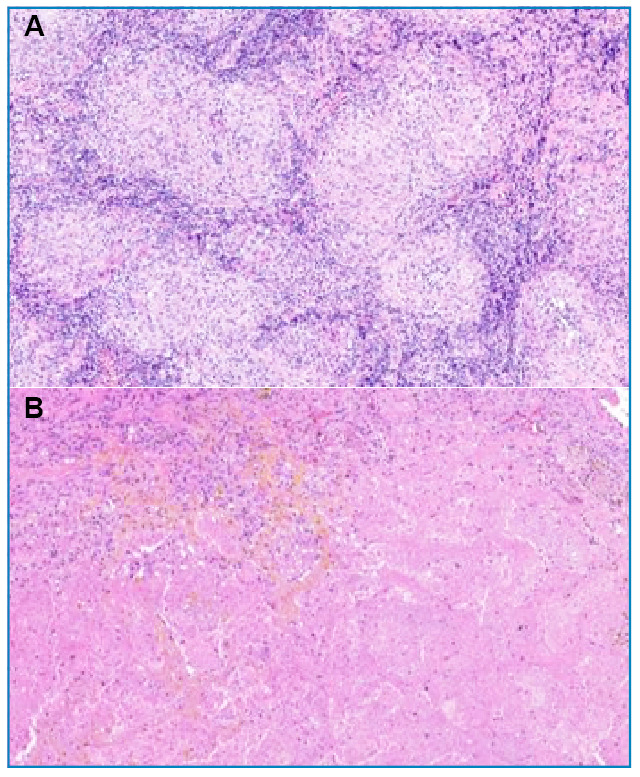
**(A)** Paraaortic lymph node biopsy, presenting gigantocellular reaction with foci of caseous necrosis associated with a lymphocytic inflammatory infiltrate. Hematoxylin-eosin, 40x magnification. **(B)** Left upper lobe biopsy of the lung, showing caseous necrosis, adjacent epithelioid histiocytes, organized alveolar pneumonia, alveolar septal/interstitial fibrosis, central acinar emphysema and alveolar exudate macrophages. Hematoxylin-eosin, 40x magnification.

The biopsy of the para-aortic lymph node showed foci of caseous necrosis associated with lymphocytic inflammatory infiltrates (Figure 2A). Tests for fungi (periodic acid-Schiff and Grocott methenamine-silver staining) yielded negative results. Neoplasia was not observed. A left upper lobe lung biopsy showed caseous necrosis, adjacent epithelioid histiocytes, organized alveolar pneumonia, alveolar septal or interstitial fibrosis, centriacinar emphysema, and alveolar exudate macrophages (Figure 2B). The specimen was negative for fungi and positive for acid-fast bacilli, and polymerase chain reaction (PCR)-positive for *M. tuberculosis.* No neoplasia was found. The final histopathological diagnosis was tuberculosis. The clinical and laboratory data suggested a case of ITP triggered by tuberculosis.

Myelography showed hypercellular bone marrow with erythroid and megakaryocytic hyperplasia, preserved cell maturation, and no anomalous cells. Since other causes of thrombocytopenia had been ruled out, and the platelet count response was satisfactory and progressively increased, reaching 120,000/µL in the first 15 days of treatment, ITP was diagnosed.

The patient was discharged in November 2019, and treatment for tuberculosis was initiated with a rifampicin/isoniazid/pyrazinamide/ethambutol (RIPE) regimen. Corticosteroids were simultaneously tapered off, and 50 mg/day of the immunosuppressant azathioprine was introduced because of fluctuations in platelet count while the corticosteroids were being tapered off. During that period, the patient became asymptomatic with no bleeding and platelet count >100,000/μl. In February 2020, three months after discontinuing corticosteroid use, the patient presented with bleeding gums and platelet count of 10,000/μl. The patient was then readmitted to the hospital. Prednisone at 80 mg/day and azathioprine at 100 mg/day were administered. RIPE was maintained, with platelet count >150,000/μl and mucosal and skin bleeding resolving in March.

**FIGURE 3:**
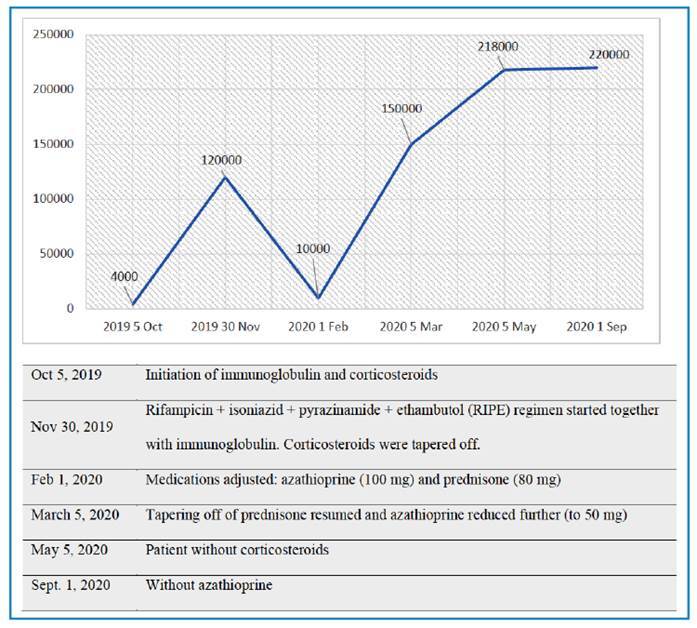
Graph showing platelet count per microliter at hospital admission and throughout treatment.

At the end of March 2020, the patient was discharged for the second time, and weekly tapering of corticosteroids was initiated again. Azathioprine was reduced to 50 mg/day, platelet count was 150,000/µL and there was no bleeding. Outpatient tuberculosis treatment ended in May 2020, with no more reported bleeding and platelet counts being consistently normal in June (218,000/µL) and September 2020 (219,000/µL). By then both corticosteroids and azathioprine had been discontinued (Figure 3).

## DISCUSSION

The pathophysiology of tuberculosis as a cause of ITP is yet to be fully elucidated. The most widely accepted hypothesis is that *M. tuberculosis* stimulates B-lymphocyte clones to produce antibodies against autologous platelets^11^. Several other factors indirectly related to tuberculosis have also been described as possible causes of thrombocytopenia, including the use of drugs for the treatment of tuberculosis, latent ITP compounded by an infection (tuberculosis or otherwise), hypersplenism, and occupation of the bone marrow space by fibrosis or granuloma^1^. The presence of any of these conditions complicates the diagnosis of tuberculosis-triggered ITP.

The diagnosis of ITP depends on ruling out other causes of thrombocytopenia. Other potentially associated diseases should also be investigated^11^. ITP that is secondary to infections such as *M. tuberculosis*, as in the present case, must be considered. Investigations are usually based on tests for tuberculosis infection, including the tuberculin purified protein derivative (PPD) (Mantoux test), PCR for *M. tuberculosis* and sputum or bronchoalveolar lavage fluid smears for acid-fast bacilli. However, few cases have tested positive with these methods, as in the case reported here, in which bronchoalveolar lavage was negative. The final diagnosis was reached following a biopsy of the affected lymph node showing a giant cell reaction with foci of caseous necrosis associated with lymphocytic inflammatory infiltrate. Although the investigation for acid-resistant bacilli was negative in the lymph node specimen, the results were positive in the pulmonary biopsy specimen (left upper lobe), confirming the presence of tuberculosis^1^^,^^3^^,^^8^. In cases reported in the literature, the diagnosis was also based on biopsy, with the most common finding being the presence of granulomatous cells compatible with infection. Diagnoses can also be made through visualization of acid-fast bacilli by Ziehl-Neelsen staining^1^^,^^11^. Therefore, when ITP is concomitant with tuberculosis, a causal association between the conditions can be established.

In cases in which the association of tuberculosis with ITP was confirmed, thrombocytopenia improved within the first few days of anti-tuberculosis therapy (RIPE)^11^. Immunoglobulin therapy has been used to improve thrombocytopenia in patients with active mucosal bleeding and severe ITP. Timely use of corticosteroid and immunoglobulin treatment has yet to be established; however, it is evident that in most cases in which ITP is secondary to *M. tuberculosis*, the treatment of tuberculosis constitutes the definitive treatment for thrombocytopenia. While immunoglobulins promote a momentary increase in the number of platelets, the platelet count may still decrease if tuberculosis is not treated properly.

The patient in this case initially presented with thrombocytopenia as the primary finding. In view of the severity of thrombocytopenia, treatment was initiated with immunoglobulin and corticosteroids, and the platelet count increased in response within a few days. After the diagnosis of tuberculosis and secondary ITP was confirmed, the RIPE regimen was initiated, and azathioprine and corticosteroids were tapered off. Although platelet count initially decreased, it normalized within a few days. The platelet count remained normal after the completion of tuberculosis treatment. Weber et al. reported the case of a 22-year-old patient who presented with hypermenorrhea and petechiae due to ITP secondary to tuberculous lymphadenitis. Thrombocytopenia returned to normal after the initiation of anti-tuberculosis treatment following the failure of thrombocyte substitution and immunomodulatory treatment^11^.

Treatment for tuberculosis-triggered ITP mainly consists of the RIPE regimen. Corticosteroids and/or immunoglobulins can be used in conjunction with tuberculosis drugs to increase platelet count and improve patient outcome.

## ETHICS

This study was approved by the institution’s internal review board. The procedures involved were in accordance with Brazilian regulations and the principles of the Declaration of Helsinki.

## CONSENT

The patient whose case was reported gave his informed consent for this report to be published.
